# Analysis and Experimental Validation of Efficient Coded OFDM for an Impulsive Noise Environment

**DOI:** 10.3390/s18113667

**Published:** 2018-10-29

**Authors:** Ndéye Bineta Sarr, Basile L. Agba, François Gagnon, Hervé Boeglen, Rodolphe Vauzelle

**Affiliations:** 1 École de Technologie Supérieure (ETS), Montréal, QC H3C 1K3, Canada; francois.gagnon@etsmtl.ca; 2 XLIM Institute, University of Poitiers, 86360 Poitiers, France; herve.boeglen@univ-poitiers.fr (H.B.); rodolphe.vauzelle@univ-poitiers.fr (R.V.); 3 Institut de Recherche d’Hydro-Québec (IREQ), Varennes, QC J3X 1S1, Canada; agba.basile@ireq.ca

**Keywords:** smart grid, impulsive noise, channel coding, OFDM, software-defined radio, USRP, experiments

## Abstract

The importance of synergy with industry lies in the possibility of experimental validation of the work research results. With Software-Defined Radio (SDR) platforms, it is possible to implement a physical layer and to have tests with hardware in a real environment. In this paper, we investigate the validation of an impulsive noise resistant physical layer based on Orthogonal Frequency Division Multiplexing (OFDM) and an interesting concatenation of forward error correcting codes: Rank metric Code (RC) and Convolutional Code (CC). We fully design and implement a new block namely RC Encoder + WiFi Mapper in GNU Radio, which acts as a forward error correcting code to mitigate impulsive noise occurring in substations. After showing by simulations that using this coding scheme is very efficient in mitigating the bursty nature of impulsive noise, we then confirm that the same performance is maintained even with various impulsive voltages and experimental scenarios, which confirms the high performance of the proposed approach.

## 1. Introduction

Intelligence in networks has been a reality for a long time. The novelty is the growing need for flexibility in electrical systems. This need finds a large part of its origin in the development of intermittent energies (wind, solar), which induce rapid variations of production. It is also reinforced by the concern to avoid capacity investments by using the flexibility of demand. To have networks capable of managing this flexibility, it is necessary to introduce new forms of intelligence. This new form of intelligence consists of placing a computer network above the grid to form a Smart Grid (SG). The functions are to ensure better control and optimization of the network and to offer value-added services. This is an evolution that has become possible because it is able to couple computer and electrical technologies in the same equipment and networks at a very low cost. Therefore, for energy companies, the use of this solution becomes unavoidable considering their number of substations and the immense extent of their electrical networks. It is within this framework that sensor networks are deployed in this context of SG since these technologies offer advantages such as a low cost of deployment. Sensor networks will allow remote control of equipment, diagnosis and maintenance of stations through a communication protocol. However, High Voltage (HV) substations are strategic points in the electrical network and are the site of numerous disturbances that can generate noise of the impulse type. This type of noise is well known in the field of electrical engineering and is a source of losses that can degrade the performance of wireless communications [1]. The existing wireless technologies such as ZigBee were not designed for the power grid. They do not consider the different requirements of the industrial environments and cannot guarantee an acceptable quality of communication and good performance [2]. Industrial domains are not taken into account in the design of existing standards because their constraints have particular characteristics (reliability, real-time, multi-path phenomena, low power consumption, safety, etc.). Many previous research works have proposed precise impulsive noise models [3,4,5,6,7,8,9]. For this purpose, in [10], the author suggested a new impulsive noise mitigation technique derived from the application of block-based Compressed Sensing (CS). A semi-analytical error-rate performance was also studied, which demonstrated that the proposed approach is efficient in CS application for impulsive noise detection. Furthermore, Coded-Orthogonal Frequency Division Multiplexing (C-OFDM) systems have been widely used for impulsive noise cancellation. In [11], the authors designed a low-density parity check coded OFDM system applicable to the impulsive noise channel. The results of the proposed approach demonstrated precisely that the performances are much better than the conventional systems. Besides, Xin et al. introduced in [12] an efficient impulsive noise estimation algorithm based on the Alternating Direction Method of Multipliers (ADMM) for OFDM systems using Quadrature Amplitude Modulation (QAM). Simulation results have shown that the proposed method can achieve bit error rate performance improvements at reduced computational complexities compared with the conventional techniques.

In our recent work [13], we proposed a resistant physical layer based on the concatenation of Rank metric Code (RC) and Convolutional Code (CC) with OFDM. We have used this model to compare different signalling schemes on this channel [13]. The results showed that our approach is robust and efficient compared to other systems. However, the obtained results are based solely on simulations. Moreover, in the literature, there does not exist experimental results for impulsive noise suppression that can be used as a basis for comparison. Several works have been proposed, but the results are only based on simulations. To validate the proposed physical layer, it is essential to compare the theoretical results to the experimental ones. Indeed, in recent years, Software-Defined Radios (SDRs) have drastically changed the way that research and experimental works are done in the wireless arena. Their advantages over traditional hardware-based radios are that significant aspects of the communication stack are implemented in software programs [14], while in hardware radios, the lower layers are realized in hardware, making their use extremely constrained when modifications on physical or medium access layers are required. Thus, with SDRs, most of the radio functionality and signal processing are implemented in software that runs on a computer, and the only task of the hardware is taking care of functions, such as transmissions and reception of the signal [15]. We now propose a combined analysis and experimental validation of the best coding schemes with an appropriate OFDM signal. The objective is to come up with an optimized overall physical layer to ensure better communication in substations. For this purpose, we use the GNU’s Not Unix Radio (GNU Radio) platform, which is presently the open-source reference apparatus for academic work and wireless research [15,16,17,18,19] allowing the researcher to implement signal processing blocks with no additional hardware requirements. We implement a new block namely “RC Encoder + WiFi Mapper” to perform error correction. It is based on the implementation of *gr-ieee* 802.11 proposed by Bloessl et al. [20,21]. The main contributions of this paper are summarized as follows:

- Implementation of a new block performing forward error correction code based on the RC code and convolutional encoding in GNU Radio.
- Setup design and impulsive noise measurements in a controlled laboratory condition: this is a new method using the GNU Radio instead of an oscilloscope as proposed in other laboratory tests.
- Over the air experiments in an impulsive noise environment in a laboratory: several scenarios are made up including Line-Of-Sight (LOS) and Non-Line-Of-Sight (NLOS) situations by also considering the impulsive noise level, the distance between the emitter, the receiver and the source.

The rest of this paper is organized as follows. Section 2 presents an overview of the system. It first shows the simulation chain before describing the transmitter and the receiver designed in GNU Radio. The setup for impulsive noise measurements in a controlled laboratory is detailed and some results are also presented in Section 3. The obtained results with this specimen demonstrate that this new method can reproduce the impulsive noise accurately. Section 4 provides the specifications and the measurement scenarios. In Section 5, we present the performance analysis of the obtained results. It first details the network performance by examining the overall rate of traffic and the average delay, which shows that the LOS situation presents better performance than the NLOS configuration. After that, the Bit Error Rate (BER) performance in the presence of impulsive noise obtained by experiments is presented. The performances obtained in this section are globally satisfactory even if we can notice a little degradation in some cases. A comparison between the simulation results shown in [13] and the experimental results is also overviewed in this section, which confirms that the same performances are maintained with several impulsive noise level voltages. Finally, the conclusion and perspectives are given in the last section.

## 2. System Overview

In this section, we present the physical layer overview. It describes the main elements that constitute the proposed system.

### 2.1. Description

**Figure 1:**
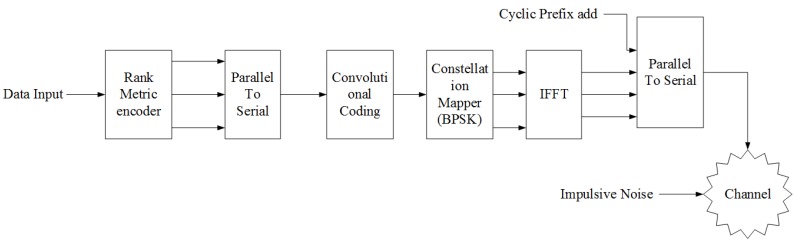
The physical layer overview.

The proposed physical layer [13] consists of a C-OFDM-based RC code concatenated with a CC scheme, as depicted in Figure 1. RC coding has been thoroughly studied in the literature in coding theory, but used now in communication systems to mitigate impulsive noise. Since the noise present in power substations appears as burst errors, this kind of scheme is well suited. However, it has been demonstrated that the background noise considered as Gaussian noise is also present in this environment. In order to cancel the isolated noise, the use of a CC becomes essential.

#### 2.1.1. Rank Metric Mapping

In the multi-carrier transmission system, the signals can be “naturally” represented in a matrix system, where each column is used to generate an OFDM symbol. Let us define a vector B of elements in the finite field $F_{q}^{N}$, B = $\left(b_{1},b_{2},\ldots ,b_{n}\right)$ the signal to be sent. $F_{q}^{N}$ can be considered as a vector over the space $F_{q}$. Let us call A a base of $F_{q}^{N}$ over $F_{q}$. We can represent vector B as a matrix *B* with entries in the finite field $F_{q}$ by decomposing the elements $b_{i}$ of B following the base A.

(1) $$B=\left[\begin{array}{c} \left[\begin{array}{c} b_{11}\\ b_{21}\\ \vdots \\ b_{N1} \end{array}\right]\left[\begin{array}{c} b_{12}\\ b_{22}\\ \vdots \\ b_{N2} \end{array}\right]\ldots \left[\begin{array}{c} b_{1n}\\ b_{2n}\\ \vdots \\ b_{Nn} \end{array}\right] \end{array}\right]$$

Since a binary modulation will be used, *q* = 2. By using an RC defined on $F_{q}^{N}$, a code vector can be expressed as follows:

(2) $$v=x\times G=(v_{1},v_{2},\ldots ,v_{n})$$

where *x* is the message to sent and *G* is the generator matrix. Thus, *v* can be represented as a matrix in the finite field $F_{2}$ as follows:

(3) $$V=\left(\begin{array}{cccc} v_{1,1} & v_{1,2} & \cdots & v_{1,n}\\ v_{2,1} & v_{2,2} & \cdots & v_{2,n}\\ \vdots & \vdots & \vdots & \vdots \\ v_{N,1} & v_{N,2} & \cdots & v_{N,n} \end{array}\right)$$

The elements of the matrix *V* are then transposed into a serial format in order to prepare them for the convolutional encoding. A Binary Phase Shift Keying (BPSK) is applied to ensure transmission of the coded sequence with an effective bandwidth.

#### 2.1.2. OFDM

OFDM is a multi-carrier modulation largely employed in wired systems, Power-Line Communications (PLC) [22,23] and wireless standards systems (e.g., Digital Video Broadcasting-Terrestrial (DVB-T) [24], Wimax, IEEE 802.11a/g/n/ac [25]). The high data rates and robustness to combat multi-path fading make OFDM a perfect candidate for reflective industrial environments [26]. Instead of transmitting a high-speed data stream over a single carrier, OFDM utilizes many orthogonal sub-carriers transmitted in parallel. In our case, the number of sub-carriers is *C* = 64. Each sub-carrier is modulated with a BPSK encoded matrix. An “OFDM symbol” is formed by each column of this matrix. The output denoted *F* is as follows:

(4) $$F=\left(\begin{array}{cccc} f_{1,1} & f_{1,2} & \cdots & f_{1,t}\\ f_{2,1} & f_{2,2} & \cdots & f_{2,t}\\ \vdots & \vdots & \vdots & \vdots \\ f_{s,1} & f_{s,2} & \cdots & f_{s,t} \end{array}\right)$$

where:

- $f_{ij}$ represents the tone of frequency i and time slot j,
- *s* corresponds to the sub-carriers used,
- *t* is the number of OFDM symbols sent on the channel.

### 2.2. Implementation in GNU Radio

In this section, we describe the structure of the modified *gr-ieee* 802.11 transceiver implemented in GNU Radio. The transmitter and the receiver are both constituent parts of the transceiver implementation developed by B.Bloessl et al. [21].

#### 2.2.1. The Transmitter

The structure of the transmitter is a constituent of *gr-ieee* 802.11. The difference between the recent implementation of Bloessl et al. is the new block called “RC Encoder + WiFi Mapper”, otherwise the rest of the blocks remains the same.

**Figure 2:**
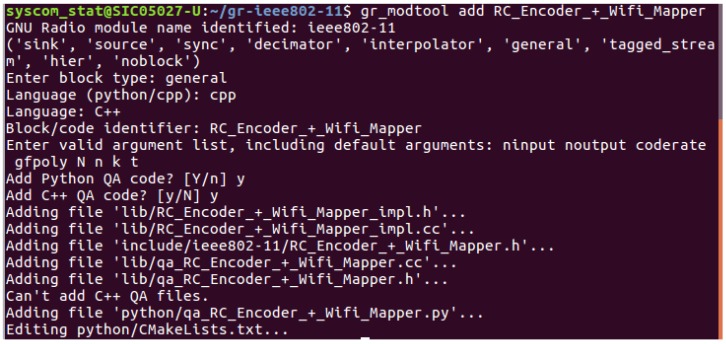
RC Encoder + WiFi Mapper block adding in *gr-ieee* 802.11.

RC Encoder + WiFi Mapper implementation: The novelty in this structure is the new block that is created for RC encoding and CC encoding. First, we recover the old implementation of *gr-ieee* 802.11. Following the steps of the building, which can be found in [27], we create a new block by using *gr-modtool add* and the different arguments linked to the blocks as depicted in Figure 2.

The various parameters associated with this block are also identified:

- ninput corresponds to the data from the Medium Access Layer (MAC). They can be in a message or a bit format.
- noutput represents the encoded data after convolutional encoding.
- coderate is the coding rate of the convolutional encoder and = 1/2.
- gfpoly corresponds to the primitive polynomial of the rank metric encoder. Since we use *N* = 16, the generator is as follows:
  (5) $$p\left(\alpha \right)=\alpha^{16}+\alpha^{12}+\alpha^{3}+\alpha +1$$
- *N* is the size of the Galois field.
- *t* represents the power of correction.

After this block of error correction code, we have the other blocks such as packet header generator, which generates the header of the frame, including the signal and service fields. The header and the remaining frame are both BPSK modulated using chunks to symbols. After that, an OFDM carrier allocation is performed and responsible for the aggregation of the pilot sub-carriers, and the Fast Fourier Transform (FFT) block is responsible for the inverse FFT, i.e., for the transition from the frequency to the time domain. Since the OFDM symbols will be sent to a multi-path channel, the signal at the receiver side will be distorted. One way to combat the effect of the multi-path channel such as the Inter-Symbol Interference (ISI) is to add a cyclic prefix.

#### 2.2.2. The Receiver

The receiver part of the IEEE 802.11 physical layer is designed to receive and demodulate OFDM signals. As described in [21], the OFDM receiver consists of a frame detection part, which exploits the short cyclic preamble repeated 10 times at the start of a frame. The frame is then aligned, equalized and decoded into the MAC information needed for proper delivery.

NB: Since we evaluate performance over the air, the transmitter and the receiver used are interfaced with USRP radios. At the receiver side, recall that the last block was the decoding of the frame. However, this decoding is only performed by the Viterbi decoder. After that, we use Wireshark connector to save the information and performs MATLAB processing for the haul decoding of the rank metric.

## 3. Impulsive Noise Measurements

Generally, in the signal processing domain, there are at least two types of noise: the white Gaussian noise and impulsive noise [28]. Currently, the statistical behaviour of Gaussian noise is well known. In contrast, impulse noise is a non-Gaussian random process that has a strongly structured form due to its impulsive nature. It often has non-stationary characteristics such as the appearance of random pulses over time with high levels of interference [29]. For measuring the electromagnetic pulses radiated by partial discharges, several experiments have been conducted with antennas [30,31,32]. For example, in [33], Shan et al. presented a procedure for measuring impulsive noise in electricity substations with three antennas covering the range 100 MHz–6 GHz. However, since the measurement in power lines is not always possible due to several constraints such as the climatic conditions and the availability of qualified personnel, another alternative has been designed to evaluate the partial discharges. It consists of tests in the laboratory that are done using a Tesla coil or generator bar. In [34,35], the authors designed a setup for tests in the laboratory, and the results showed that it is possible to use these devices to generate partial discharges or gap noises.

### 3.1. Description of the Setup

Measuring impulsive noise becomes essential for evaluating the characteristics that are considered in the design of a communication system. For the laboratory measurements, the main components are a generator bar and a Universal Software Radio Peripheral (URSP) radio. The setup is described in the following.

**Figure 3:**
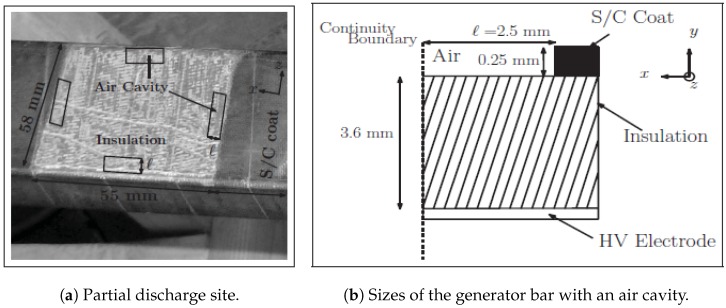
The plane of a generator bar with the designed air cavities [28].

- The generator bar is used in controlled laboratory conditions under different voltages. Hydro-Québec researchers use this device to evaluate the partial discharges localization on the voltage phase. In the middle of the bar, the insulation is covered by a conductor made with epoxy-mica insulated by a shield, as depicted in Figure 3. The principal characteristics of the dimensions are summarized in Table 1.
  When a high voltage generator (1–18 kV) is connected, an electromagnetic field is emitted radially by the conductor that the isolator tries to isolate. The generator bar is an excellent candidate for partial discharge generation, and it controls the power supply accurately.
- USRP N210 [29] connected to an antenna is used for recovering the signal from the generator bar. Indeed, we implement some blocks to save the measures issued from the impulsive noise generator. More information on the functioning of the USRP are given in Section 4.

### 3.2. Results

**Figure 4:**
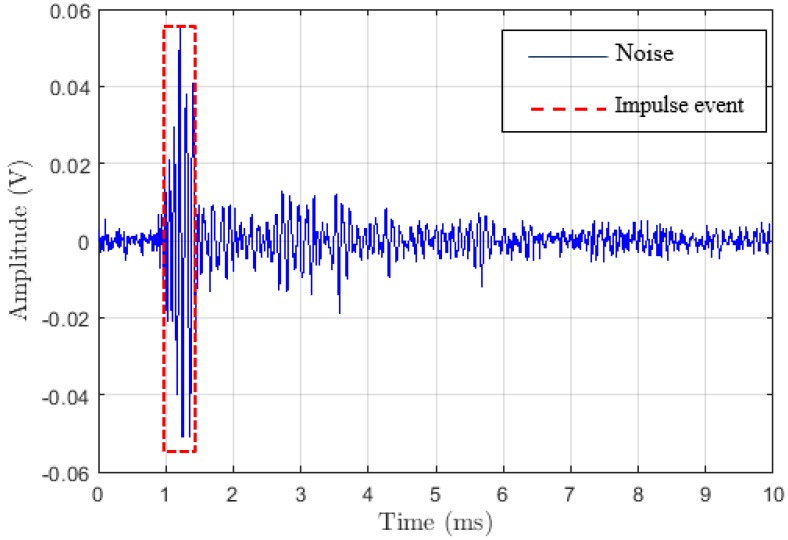
Impulsive noise from the generator bar at 16 kV.

Figure 4 represents an example of measurements made with the generator bar at 16 kV. It corresponds to the plot of the noise level (amplitude) versus the time in milliseconds. In this figure, we notice that a short duration and high amplitude characterize the impulsive event, which is highlighted by the red-dashed contour. As can be seen, the alternative method for measuring partial discharges is also accurate. It represents the impulse event well and the background noise compared to the results from measurements obtained in a power substation [2].

## 4. Experimental Planning and Test Bed Design

This section deals with the description of the hardware and software specifications needed in the test bed. We also overview the measurement scenarios where we conduct the experiments.

### 4.1. Specifications

We now present a brief description of the setup used for the experimental tests.

#### 4.1.1. Hardware

The materials necessary to perform the experimental measurements include a USRP N210 from Ettus Research, an antenna, etc. A brief description of each of these components is given below.

- USRP N210: It is an SDR product that enables designing and implementing software radio systems. The USRP N210 presents high-dynamic range processing capability and high bandwidth [36]. It is compatible with a wide range of Radio Frequency (RF) modules called daughter-boards. In receiving mode, the samples acquired by the daughter card are scanned by the Analogue-to-Digital Converter (ADC), then converted to the desired intermediate frequency (IF). Finally, the sequence of samples is multiplied by a sine and a cosine to obtain respectively the two components I and Q. The motherboard is connected to a daughter-board allowing the transmission and reception of analogue RF signals. On its daughter-boards, the signal is filtered, amplified and put on a baseband frequency that depends on the IF band of the board and the frequency of the local oscillator. These cards also include basic Rx/Tx cards without conversion frequency or filtering. The two daughter-boards used in this paper are RFX 2400 and SBX. These cards can operate in duplex mode, integral for simultaneous transmission and reception. They offer a bandwidth up to 50 MHz and cover the 2.4-GHz band.
- Antenna: The antenna used is the *ANT*-2.4-*CW*-*HW* model by Linx. It belongs to the HW series 1/2-*wave* center-fed dipole antennas. It is an omnidirectional antenna in wide cover rubber. It uses a horizontal polarization and is connected to the USRP via a SubMiniature Version A (SMA) connector. The characteristics of the antenna are among others a frequency range between 2300 and 2600 MHz, a gain of 3.2 dBi and a maximum power of 50 W.
- Generator bar: To generate the impulsive noise in the laboratory, we use the generator bar as described in Section 3.

#### 4.1.2. Software

The different components are listed in Table 2.

### 4.2. Measurements Scenarios

This section discusses the different scenarios used to perform the measurements. It is divided into two scenarios that are described in the following paragraphs.

#### 4.2.1. Experimental Scenario 1

**Figure 5:**
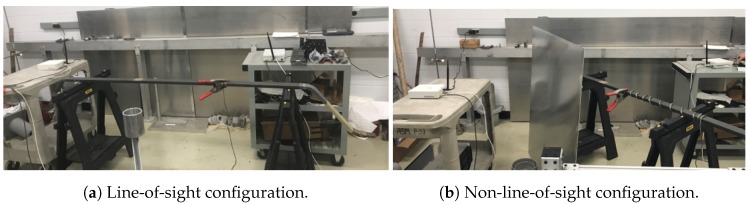
Measurement scenarios.

For the first experimental scenario, we consider an LOS situation as depicted in Figure 5a. We consider two configurations in this situation.

- The impulsive noise source is placed at equal distance from the emitter and the receiver, which is 1 m. The distance between the two USRPs is 2 m.
- In the second case, we place the emitter at 1.90 m from the source, and the receiver is at 1.80 m from the source.

For all the configurations, we generate four types of voltage for impulsive noise from 12 kV–18 kV. We limit the maximum to 18 kV to avoid the risk of breakdown.

#### 4.2.2. Experimental Scenario 2

The second experimental scenario consists of NLOS situations. It is very similar to the first case except for one detail. We use a metallic reflector as depicted in Figure 5b to generate multi-path phenomena.

- We consider 2 m between the two USRPs. The emitter and the receiver are at 1 m from the source.
- The second scenario consists of placing the receiver at 1.80 m from the source and the emitter at 1.90 m.

As for the LOS experiments, we evaluated four types of voltage varying from 12 kV–18 kV.

## 5. Performance Analysis

In this section, we analyse the performance results obtained in the different scenarios. First, the parameters used are defined. Secondly, tests with the proposed implementation are performed to evaluate the performance. We measure the overall traffic rate, the Packet Delivery Ratio (PDR), the average delay and the BERs using USRPs. Finally, a comparison between the simulation results obtained in [13] and the experimental ones is made.

### 5.1. Parameters Setting

In this section, we define the parameters used in the experimental tests. For this purpose, we describe in the following the transmit power and the frame format.

#### 5.1.1. Transmit Power

With the USRP, it is not possible to set the transmit power directly as a parameter. It can be configured via the usrp hardware driver, UHD gain. The output power depends on other parameters such as the frequency, the Total Harmonic Distortion (THD), which corresponds to the level of unwanted harmonics in a sinusoidal waveform. To have an approximate value of the output power, it can be measured by using a spectrum analyzer. For this purpose, in [37], the authors proposed radio frequency measurements on an SBX daughter-board. In this paper, we can notice that the output power decreases with the increasing of the carrier frequency. The UHD gain is an important parameter; nonetheless, its increment has to be done carefully. Since in our test, we use a frequency of 2.4 GHz with a UHD gain of 20 dB, the corresponding output power is equal to 12.05 dBm.

#### 5.1.2. Frame Format

The frame that we consider in our simulations is composed of three main parts and can be explained as follows.

1. RadioType header: This contains all the radio information and has a length of 17 bytes. It is composed of the fields represented in Table 3.
2. Frame information with a length of 28 bytes is composed of a Frame Control Field (FCF) and Logical Link Control (LLC), as presented in Table 4. LLC contains addressing information consisting of two fields: the Destination Service Access Point (DSAP) and the Source Service Access Point (SSAP), as well as a control field.
3. Data correspond to the useful information that will be encoded. Its length is 316 bytes.

We run simulations by considering the two scenarios mentioned above. We use the same traffic load, which corresponds to one packet sent every 300 ms. The duration time of each simulation is approximately 120 s.

### 5.2. Network Performance Analysis

In this section, we examine the overall traffic rate, the PDR and the average delay of the packets. The following annotations are used:

- ${\left\{X\right\}}_{LOS1}$ corresponds to the case of the LOS situation and when the emitter and the receiver are both positioned at 1 m from the noise source.
- ${\left\{X\right\}}_{NLOS1}$ is the case of the NLOS situation and when the transmitter and the receiver are both located at 1 m from the impulse source.
- ${\left\{X\right\}}_{LOS2}$ represents the case of the LOS situation and when the emitter is placed at 1.90 m from the source and the receiver at 1.80 m.
- ${\left\{X\right\}}_{NLOS2}$ corresponds to the case of the LOS situation and when the emitter is positioned at 1.90 m from the source and the receiver at 1.80 m.

where {X} is either μ or PDR.

#### 5.2.1. Traffic Rate

We compute the primary Input/Output (IO) graphics from Wireshark in different situations. The IO graphs identify the overall rate of traffic seen in the capture files. The X axis corresponds to the time in seconds, and the Y axis is the packets per second.

**Figure 6:**
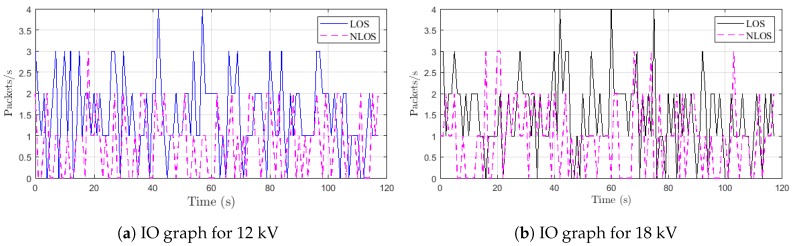
Overall rate of traffic for 12 and 18 kV when the emitter and the receiver are both placed at 1 m from the source.

Two examples of the results are depicted in Figure 6. The traffic for 12 kV in the first situation is plotted in Figure 6a, while Figure 6b represents the traffic for 18 kV. The first remark is that we receive more packets in the LOS situation than NLOS for the two configurations. When we analyze the results in Figure 6, we can note that the voltage does not affect the traffic of the received packets considerably. However, we have a degradation of performance when we are in an NLOS situation. This means that the traffic of the LOS environment can be controlled and managed, whereas it is not possible for NLOS because of the events’ randomness.

In Table 5, we evaluate LOS and NLOS traffic averages for both configurations. From this table, we notice that the average $\mu_{LOS1}$ is around 1.5 regardless of the voltage level, while in NLOS, the average $\mu_{NLOS1}$ is approximately one. We derive Δ, which is the difference between the average in LOS and NLOS. This difference $\Delta_{1}$ is equal to 0.5. However, for the second situation, we observe that the average traffic is roughly similar in LOS and NLOS, i.e., $\mu_{LOS2}$≈$\mu_{NLOS2}$. This is well proven by the difference $\Delta_{2}$, which is approximately equal to 0.1.

In order to analyze the traffic in depth, we now compute the PDR, which is the ratio between the received packets and the emitted ones. The following relation defines the PDR:

(6) $$PDR=\frac{\sum P_{r}}{\sum P_{e}}$$

where $P_{r}$ and $P_{e}$ are the received and the emitted packets, respectively. The obtained results are summarized in Table 6.

The information in Table 6 confirms the analysis made with the overall traffic rate. We can note that we received more packets for the LOS than the NLOS situation. Another remark is that we have approximately the same delivery ratio when considering the voltage. As can be seen, the $PDR_{mean_{LOS}}$ is ≈ 0.4, which means that 60% of packets are lost. However, for NLOS situation, 70% of packets are lost, which means that $PDR_{mean_{NLOS}}$ is ≈ 0.3. This was expected because when we use a transmission power of 12 dBm, it is demonstrated that with the SDR, the PDR is ≈ 0.5 in normal conditions, i.e., with a low length of packets (95 bytes) and a BPSK modulated with a rate of 1/2. To improve the PDR, we should increase the transmission power.

#### 5.2.2. Average Delay

In this section, we compute the average delay versus the voltage for the overall situations. With Wireshark capture files, we have the information about the delta time from the previously captured frame and the time reference since the first frame. The average delay defines the average sum of the difference delay of each data packet received by the sink and the time the source sends a data packet. It is calculated as follows:

(7) $$A_{d}=\frac{\sum_{i=1}^{N_{P}}\left(T_{ri}-T_{ei}\right)}{N_{P}}$$

where $T_{ri}$ is the time when a packet *i* is received by the sink, $T_{ei}$ the time when a packet *i* is sent by the source and $N_{P}$ corresponds to the total number of the received packets. In this case, we limit $N_{P}$ to 100.

**Figure 7:**
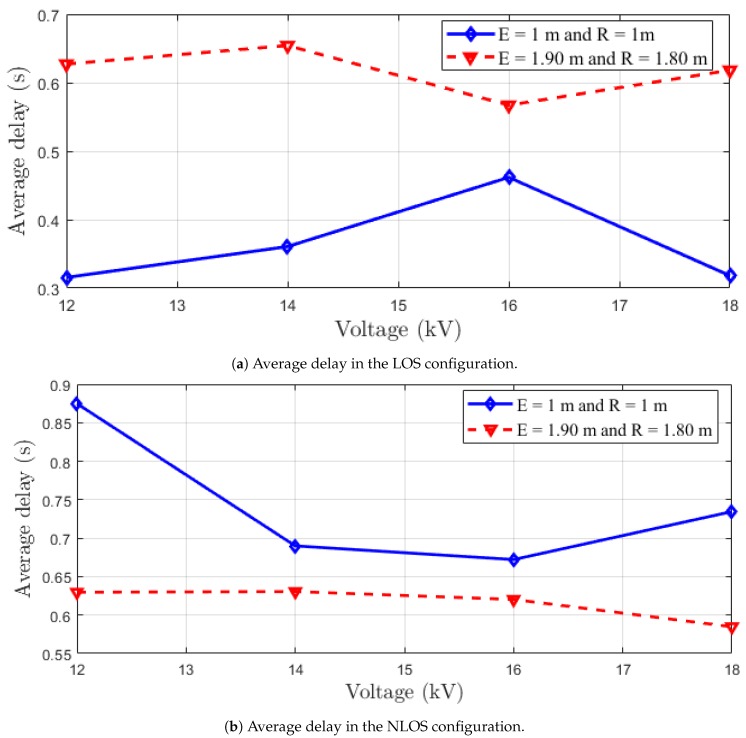
Average delay performance versus the voltage for all configurations.

The obtained results are depicted in Figure 7. The results for LOS configurations are in Figure 7a. As can be seen, the average delay increases with the distance. The average delay for the LOS configuration is equal to 0.4 s. We can also note that the 16 kV have the most significant delay in the first case and the lowest one when we increase the distance. Another remark is that the average delay is less important for the LOS situation than the NLOS situation, as expected. This can be explained by the fact that in the NLOS situation, we have the delay due to the multi-path phenomenon.

The results for the NLOS configuration are depicted in Figure 7b. However, we can note that when the distance increases, the delay decreases, contrary to the LOS situation. The worst delay is obtained when the voltage is equal to 12 kV for 2 m between the transmitter and the receiver. This is expected and can be explained by the IO graphics in which the received packets are more important in LOS than those obtained in NLOS. The average delay for the NLOS situation in all configurations is equal to 0.67 s.

### 5.3. Bit Error Rate Computation

We now present and analyze the performance results concerning bit error rate versus the energy per bit to noise power spectral density ratio (EbNo) from the different scenarios described in Section 4.

#### 5.3.1. Case 1: Emitter and Receiver Both Placed at 1 m from the Source

**Figure 8:**
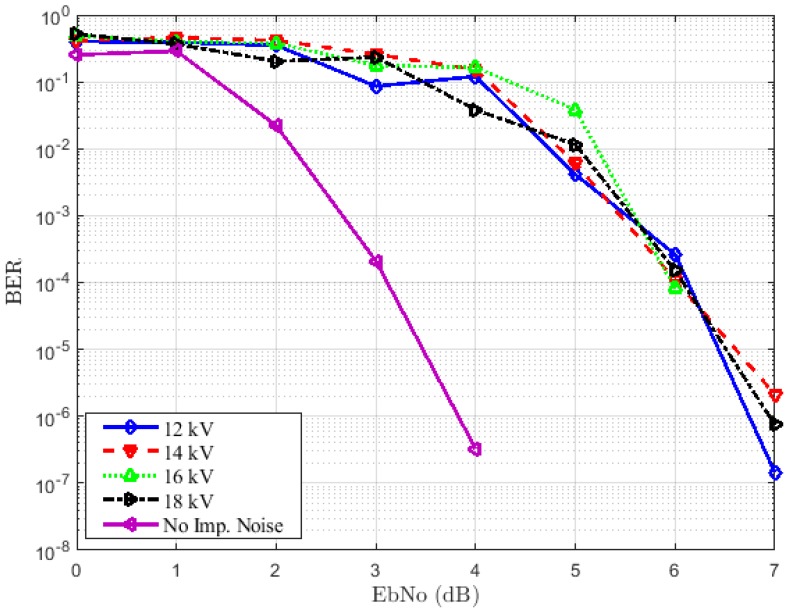
BER performance for different voltages in the LOS situation.

**Figure 9:**
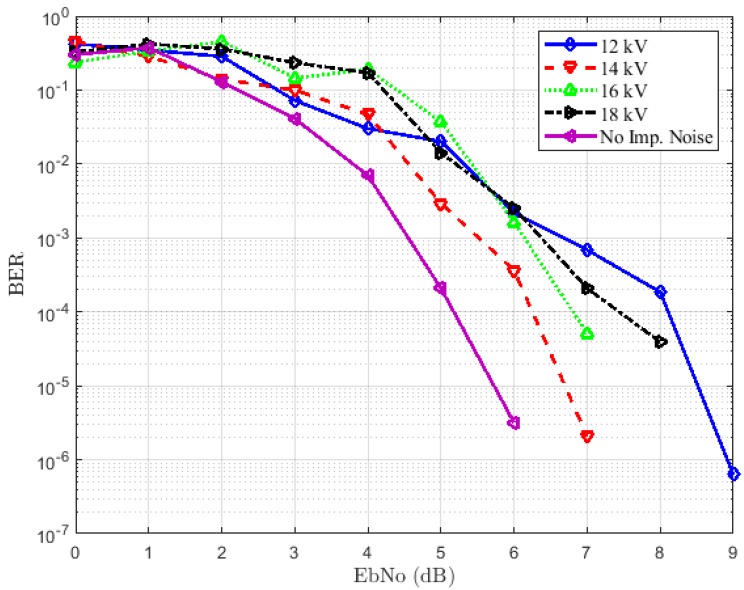
BER performance for different voltages in the NLOS situation.

The results for this first experimental scenario are depicted in Figure 8 and Figure 9 for the LOS and NLOS situations, respectively. The first remark is that we note some irregularities in the curves. This can be explained by the critical aspect of the error performance of bounded distance for the RC decoder. Indeed, considering a received word, a bounded distance either procures a codeword or affirms a failure within an agreed radius of the received word. In the first case, an error of decoding appears when the codeword produced does not correspond to the sent codeword. In addition, as can be seen in Figure 8 and Figure 9, we have a degradation of performance when the impulsive noise is present whatever the voltage used. This degradation is more important in the LOS situation than the NLOS case. In Figure 8, the voltage increase does not affect the BERs much. We have approximately the same performances both on 12 kV and 18 kV. A target BER of $10^{-4}$ is achieved at an EbNoof 3.2 dB when the channel is not affected by impulsive noise and approximately 6 dB in the presence of impulsive noise for all voltages. By comparing the results in Figure 8 and Figure 9, we can notice that the first case, i.e., the LOS case achieves the best performance. However, in Figure 9, the performance degradation is not as remarkable without impulsive noise compared to the case of the presence of impulse samples. A target BER of $10^{-4}$ is achieved at an EbNo of 5.2 dB when the impulses are not affecting the channel. The worst performances are obtained by the presence of 12 kV noise. This is expected because the channel is more affected in this case, as confirmed by the PDR, which is 0.25.

#### 5.3.2. Case 2: Emitter Placed at 1.90 m from the Source and the Receiver at 1.80 m

**Figure 10:**
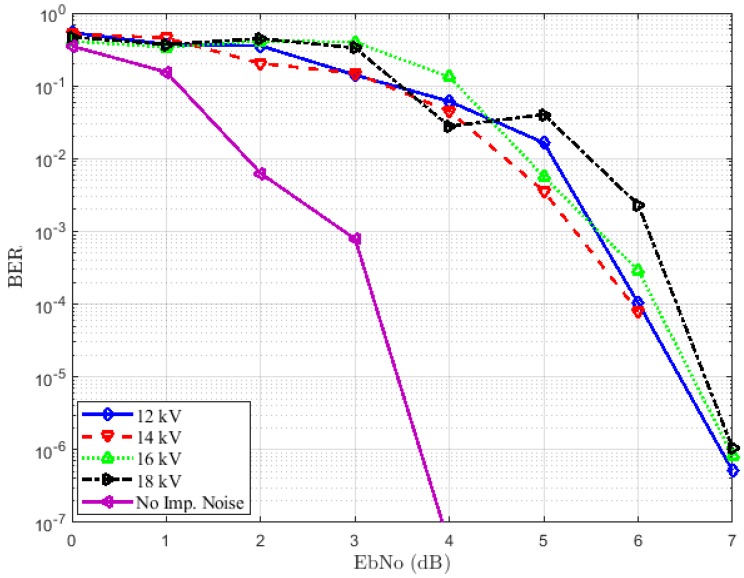
BER performance for different voltages in the LOS situation.

**Figure 11:**
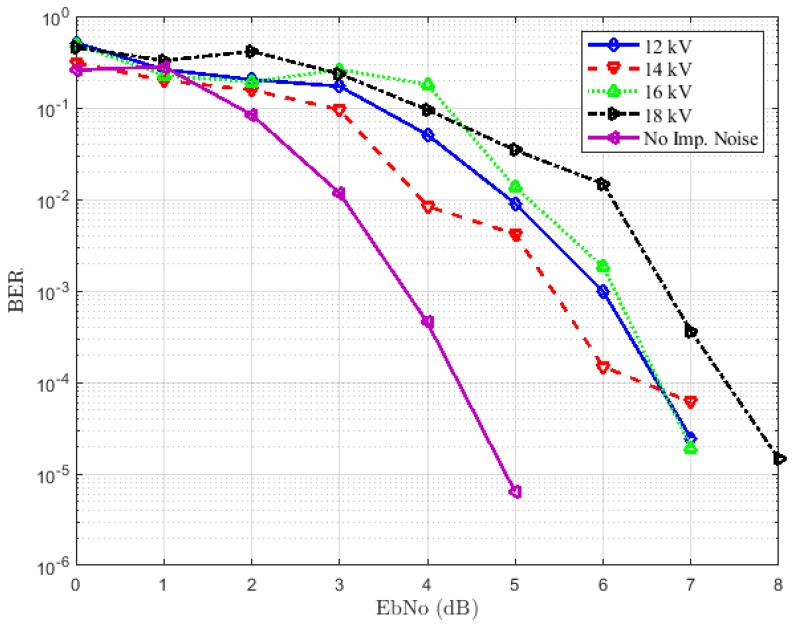
BER performance for different voltages in the NLOS situation.

We now increase the distance between the emitter and the source, as well as the receiver and the source. We have the same situations as for the first configuration, i.e., the LOS and NLOS cases. The obtained results are detailed in Figure 10 and Figure 11. By comparing the results to those obtained in the first experimental scenario, we can notice that the increase of the distance does not affect the BERs much. This remark is also valuable for the voltage. However, we can note a degradation of performance when the impulsive noise is present whatever the voltage used compared to the absence of impulsive noise. In Figure 10, a target BER of $10^{-4}$ is achieved at an EbNo of 3.2 dB and 6 dB for the 12-kV and 14-kV noise samples, respectively. When the channel is affected by 16-kV and 18-kV noise, the target BER of $10^{-4}$ is approximately 6.2 and 6.5 dB, respectively. Nevertheless, without an impulsive noise, a target BER of $10^{-4}$ is achieved at an EbNo of 4.4 dB, as depicted in Figure 11. The degradation is approximately 2.1, 1.6, 2.1 and 3.1 dB for 12-, 14-, 16- and 18-kV impulsive noise, respectively.

### 5.4. Comparison between Simulations and Measurements

In this section, we examine the validity of our proposed physical layer. In [13], we performed simulations, and in this paper, we have evaluated the performances empirically in a controlled laboratory. To validate or reject the results, we first set the difference between the simulation results and the empirical ones. Since in our experiments, we have evaluated two types of distances and four voltages, we consider the BER for LOS as the mean of all configurations, and the same procedure is applied for the NLOS situation. The results are presented in Table 7. After that, a Kolmogorov-Smirnov (K-S) test is done using the kstest2 function of MATLAB. The two-sample K-S test is a non-parametric hypothesis test that evaluates the difference between the distributions of the two sample data vectors over the range of *x* in each dataset. Mathematically, it can be written as follows:

(8) $$T_{K-S}=\underset{x}{\sup}\left(F_{1}\left(x\right)-F_{2}\left(x\right)\right)$$

where $F_{1}\left(x\right)$ and $F_{2}\left(x\right)$ correspond to the proportion of $x_{1}$ and $x_{2}$ values less than or equal to *x*, respectively.

In Table 7, we can notice that the difference between the simulation results and the experimental results for a target BER of $10^{-4}$ is quite small for the LOS and NLOS cases. We have a difference of 1.1 dB for the LOS situation, whereas for NLOS, it is 0.7 dB. These results validate the implementation in GNU Radio and confirm that our proposed approach is very efficient in mitigating the bursty nature of impulsive noise while having quite a low level of complexity. The K-S test is also another confirmation of this assertion. For LOS and NLOS, the return value of zero indicates that the test does not reject the null hypothesis at a 5% significance level. In other words, the results of the K-S test validate that the measurement results and the simulation results come from the same distribution at a confidence interval of 95%.

## 6. Conclusions

This paper investigates the validation of an impulsive noise-resistant physical layer using GNU Radio and USRP. For this purpose, a new block, namely RC Encoder + WiFi Mapper, is designed and implemented in GNU Radio. It is based on the former implementation of *gr-ieee* 801.11 made by Bloessl et al. Under different scenarios in a controlled laboratory, we have done several experiments to validate the proposed approach in the presence of impulsive noise. Using several metrics such as the packet delivery ratio, the average delay and the BER, the performances of the system are evaluated. The obtained results are globally satisfactory and confirm the assertion made in [13]. However, based on the PDR results, we recommend using a transmission power of at least 15 dBm in order to benefit the maximum packet delivery. As perspectives, it may be interesting to do other tests by further increasing the distance between the emitter and the source, as well as the receiver and the source and a voltage above 18 kV if possible. As stated in our Introduction, only a few studies are related to impulsive noise reduction techniques. For this purpose, it would also be interesting to implement in a future work two of the approaches proposed in [6,7,8,9]. This will allow us to compare our experimental results.

## Figures and Tables

**Table 1:** Characteristics of the air cavity.

Dimensions	Air Cavity	Dielectric
Height (mm)	0.25	3.6
Width (mm)	2.5	55
Depth (mm)	2	58
Relative permittivity ($\epsilon_{r}$)	1	4
Conductivity ($\sigma_{i}$)	0	$2.8\times 10^{-9}$

**Table 2:** Overview of the software used to verify the transceiver system.

Components	Types
Operating system	Ubuntu V. 16.4 LTS
CPU	Intel Core i7-2600
GNU Radio	Version 3.7.10
Matlab	R2016a
Wireshark	Version 2.4.5.

**Table 3:** RadioType header subframe format.

Fields Types	Components	Values (Bytes)
	Header revision	1
Header	Header pad	1
	Header length	2
Flag	Present flags	4
	Flags	1
Rate	Data rate	1
Channel	Channel frequency	2
	Channel flags	2
	SSI signal	1
Signal	SSI noise	1
	Antenna	1

**Table 4:** FCF and LLC sub-frame format.

Fields Types	Components	Values (Bytes)
Frame Control Field	Type/Subtype	1
	Version	1
Duration ID	Duration	2
	Destination address	6
Addresses	Source address	6
	Broadcast address	6
Sequence control	Fragment number	1
	Sequence number	1
	DSAP	1
Logical Link Control	SSAP	1
	Control field	2

**Table 5:** Mean of the overall rate of traffic for all voltages in different configurations.

	Voltages			
**Mean**	**12 kV**	**14 kV**	**16 kV**	**18 kV**
$\mu_{LOS1}$	1.5	1.42	1.31	1.48
$\mu_{NLOS1}$	1.05	0.9	0.98	0.95
$\Delta_{1}$	0.45	0.52	0.33	0.53
$\mu_{LOS2}$	1.025	1.09	1.16	1.05
$\mu_{NLOS2}$	0.84	1.08	1.05	1.14
$\Delta_{2}$	0.18	0.01	0.11	-0.09

**Table 6:** Packet delivery ratio for all voltages in different configurations.

	Voltages			
**PDR**	**12 kV**	**14 kV**	**16 kV**	**18 kV**
$PDR_{LOS1}$	0.45	0.43	0.39	0.44
$PDR_{LOS2}$	0.31	0.33	0.35	0.32
$PDR_{mean_{LOS}}$	0.38	0.38	0.37	0.38
$PDR_{NLOS1}$	0.25	0.3	0.29	0.28
$PDR_{NLOS2}$	0.32	0.32	0.31	0.34
$PDR_{mean_{NLOS}}$	0.28	0.31	0.3	0.31

**Table 7:** Summary of the performance for a target BER of $10^{-4}$.

	LOS	NLOS
Simulations	5 dB	6.2 dB
Measurements	6.1 dB	6.9 dB
$\Delta_{SM}$	1.1 dB	0.7dB

## Abbreviations

The following abbreviations are used in this manuscript:

ADC	Analogue to Digital Converter
ADMM	Alternating Direction Method of Multipliers
BER	Bit Error Rate
BPSK	Binary Phase Shift Keying
CC	Convolutional Code
CS	Compressed Sensing
DSAP	Destination Service Access Point
DVB-T	Digital Video Broadcasting-Terrestrial
FCF	Frame Field Control
FFT	Fast Fourier Transform
HV	High Voltage
IO	Input Output
ISI	Inter-Symbol Interference
QAM	Quadrature Amplitude Modulation
LLC	Logical Link Control
LOS	Line-Of-Sight
MAC	Medium Access layer
NLOS	Non-Line-Of-Sight
OFDM	Orthogonal Frequency Division Multiplexing
PDR	Packet Deliver Ratio
PLC	Power Line Communications
RC	Rank Metric
UHD	USRP Hardware Driver
USRP	Universal Software Radio Peripheral
SDR	Software-Defined Radio
SG	Smart Grid
SMA	SubMiniature Version A
SSAP	Source Service Access Point
THD	Total Harmonic Distortion
